# The understanding of Parkinson's disease through genetics and new therapies

**DOI:** 10.1002/brb3.2577

**Published:** 2022-04-21

**Authors:** Olivier Uwishema, Helen Onyeaka, Rawa Badri, Ayşe Nazlı Yücel, Ahmet Kayhan Korkusuz, Abayomi Oyeyemi Ajagbe, Amro Abuleil, Céline Chaaya, Baraa H. M. Alhendawi, Elie Chalhoub

**Affiliations:** ^1^ Department of Research and Education Oli Health Magazine Organization Kigali Rwanda; ^2^ Department of Project and Education Clinton Global Initiative University New York USA; ^3^ Department of General Medicine Faculty of Medicine Karadeniz Technical University Trabzon Turkey; ^4^ Department of Chemical Engineering School of Chemical Engineering University of Birmingham Edgbaston Birmingham UK; ^5^ Department of Research Mycetoma Research Centre Khartoum Sudan; ^6^ Department of Medicine Faculty of Medicine University of Khartoum Khartoum Sudan; ^7^ Department of General Medicine Faculty of Medicine Ankara Yıldırım Beyazıt University Ankara Turkey; ^8^ Department of General Medicine Faculty of Medicine Istanbul Medipol University Istanbul Turkey; ^9^ Department of Regenerative Medicine Regenerative and Restorative Medicine Research Center (REMER) Istanbul Medipol University Istanbul Turkey; ^10^ Department of Anatomy, Faculty of Basic Medical Sciences, College of Health Sciences Nile University of Nigeria Abuja Nigeria; ^11^ Department of Health Science Faculty of Health Sciences, Bristol Medical School University of Bristol Bristol UK; ^12^ Department of General Medicine Faculty of Medicine University of Saint Joseph of Beirut Beirut Lebanon; ^13^ Department of General Medicine Faculty of Medicine Al‐Quds University, Al‐Azhar branch Gaza Palestine

**Keywords:** genetic, Parkinson's disease, PD, therapy

## Abstract

**Introduction:**

Parkinson's disease is one of the progressive neurodegenerative diseases from which people suffer for years. The mechanism of this disease is associated with a decrease in the number of dopaminergic neurons in the substantia nigra (SN) while Lewy bodies are still present. As a result, both motor—ridity, tremor, and bradykinesia—and non‐motor symptoms such as anxiety and depression. Nowadays, it is well known that the cause behind Parkinson's disease is mainly environmental changes, genetic susceptibility, and toxins. Unfortunately, there is no cure for the disease but treatments. The replacement of lost neurons, α‐synuclein and apomorphine, is currently being studied for new therapies. This article focuses on history, mechanism, factors causing Parkinson's disease as well as future therapies for the cure of the diseases.

**Methodology:**

Data were collected from medical journals published on PubMed, The Lancet, Cells, and Nature Reviews Neurology databases with a predefined search strategy. All articles considering new therapies for Parkinson's disease were considered.

**Results:**

The pathophysiology of Parkinson's disease is currently reasonably understood. However, there is no definitive cure so all the treatments focus mainly on reducing or limiting the symptoms. Current treatment studies focus on genetics, replacing lost neurons, α‐synuclein and apomorphine.

**Conclusion:**

Parkinson's disease is the most common movement disorder worldwide because of the loss of dopaminergic neurons in the substantia nigra. Its symptoms include motor dysfunctions such as rigidity, tremor, and bradykinesia and non‐motor dysfunctions such as anxiety and depression. Through genetics, environmental changes and toxins analysis, it is now known that future new therapies are working on replacing lost neurons, α‐synuclein and apomorphine.

## Abstract 2

Parkinson's disease is one of the progressive neurodegenerative diseases from which people suffer for years. The mechanism of this disease is associated with a decrease in the number of dopaminergic neurons in the substantia nigra (SN) while the Lewy bodies are present.

## 1 INTRODUCTION

First described by James Parkinson in 1817, Parkinson's disease (PD) affects 1% of adults older than 60 years and is the most common serious movement disorder globally. The prevalence increases with age, with individuals before 40 rarely affected; thus, age is the leading risk factor (Hayes, 2019; Rajput, 1992s; Samii et al., 2004).

The discovery of physiopathology profoundly helped to understand the clinical features. There is a fall in the number of dopamine neurons in the substantia nigra, while Lewy bodies are present in the remaining neurons following a different pattern than normal ageing (Ascherio & Schwarzschild, 2016; Hayes, 2019; Samii et al., 2004).

This progressive neurodegenerative disease is clinically characterized by motor symptoms, such as rigidity, tremor, bradykinesia, and nonmotor symptoms, such as cognitive decline, depression, and anxiety. With the constantly ageing population, we will face more PD cases in future years.

To date, dopaminergic medication and deep brain stimulation are available to improve daily functions and quality of life, reducing the motor handicap (Lees et al., 2009). New studies are being conducted to find adequate treatment options to promote neuroprotective intervention before the onset of clinical manifestation (Samii et al., 2004). Promising therapeutic research approaches include embryonic stem cells and gene therapy.

## 2 NEW FACTORS INFLUENCING THE COURSE OF THE PARKINSON DISEASE

Environmental, genetic factors, and ethnic differences have been proposed to contribute to the ubiquity of PD pathogenesis globally (DeMaagd & Philip, 2015).

Various environmental elements such as metals, carbon monoxide, solvents, and agricultural chemicals such as paraquat have been identified to affect nigrostriatal impairment, which causes PD (Dinis‐Oliveira et al., 2006). Occupational exposure to paraquat has been disclosed to upsurge the chance of Parkinsonism by approximately threefold. Application of rotenone, an organic pesticide found typically in the seeds and stems of diverse plants, has been 2.5 times that leads to the development of PD (Pang et al., 2019). The mechanism of action rotenone which is a lipophilic mitochondrial toxin entails the inhibition of mitochondrial complex 1, upsurging reactive oxygen species and dwindling the production of adenosine triphosphate (Pang et al., 2019). Furthermore, several studies on environmental predisposing factors for PD outlined environmental factors with a highly suggestive association: head injury, anxiety, depression, beta‐blocker usage, dairy products, and traumatic brain injury (Pang et al., 2019).

From the genetic perspective, the proliferation of the *SNCA* gene via mutation has been discovered to lead to PD with penetrance upsurging with gene dosage. Recognized added monogenic causative genes for PD include *LRRK2*, *VPS35*, and *CHCHD2* causing autosomal dominant PD, and *PARKIN*, *PINK1*, *DJ‐1*, *ATP13A2*, *FBXO7*, and *PLA2G6* generating autosomal recessive PD (Pang et al., 2019).

## 3 THE SPOT OF PARKINSON'S DIAGNOSIS IN THE GENETIC ERA

A lot of environmental and genetic factors are considered as risk factors of PD. However, these factors vary from one patient to another. To illustrate, genetic susceptibility factors may alter the environmental effects (Simon et al., 2020).

PD diagnosis by genetic testing is a complicated process because the genetic variants can be found in 5%–10% of PD patients only. Inherited monogenic and idiopathic PD cases are caused by mutations in *SNCA*, *LRRK2*, and *VPS35*. The instances of early‐onset PD (age <40) are related to autosomal recessive variants like *PARKIN*, *PINK1*, and *DJ‐1*. While the late‐onset PD (age >50) is correlated with autosomal dominant variants like *LRRK2* and *GBA* (Cook et al., 2020).

The genetic diagnosis of PD focuses on the onset of age, age of diagnosis, ethnicity, and family history. The single‐gene testing is useful in specific cases, including a family history of Gaucher disease and African‐Berber ancestry; otherwise, it is not that accurate. A multigene panel identifies the PD etiology, but there are limitations in explaining the phenotype. Comprehensive genomic testing is being evaluated to diagnose PD from a genetic aspect (Cook Shukla et al., 2004).

Consequently, early PD diagnosis by genetics needs more clinical trials, proband studies, and future therapeutic strategies.

### 3.1 Toward new alleviating and curing therapies

There are modifying strategies for the future treatment approach of PD, one of them are mesenchymal stem cells that include biological nanoparticles that can act as protection in affected areas (Vilaça‐Faria et al., 2019). Moreover, the role of exosomes and mRNA as active modulators is a possible therapeutic strategy for patients with Parkinson's disease (Vilaça‐Faria et al., 2019). Additionally, phases of oscillation may be valuable in our future strategies. Therapies of dopamine in the form of dopamine agonists, monoamine oxidase B inhibitors, and levodopa significantly improve the features motor symptoms of bradykinesia and rigidity, with a strong effect upon tremor in patients of PD (Elkouzi et al., 2019). On the other hand, nondopaminergic drugs include serotoninergics, alpha 2‐adrenergic antagonists, and adenosine A2a antagonists which may benefit in relapse or progression stage of PD for motor symptoms and motor complications (Elkouzi et al., 2019). Neuromodulation may be an adjunct surgical option such as deep brain stimulation, and voltage stimulation (Chen & Chen, 2019). We should mention the importance of immunology as a method of treatment and prevention approaches like gene therapy and vaccines (Vilaça‐Faria et al., 2019). Furthermore, ongoing clinical trials could also focus on alternative medicine roles such as curcumin, Ginkgo biloba, and berberine, which already have demonstrated and proven good results in animals with PD (Vilaça‐Faria et al., 2019). Therefore, we should focus in our future studies on bioactive materials and plants as one of methodic approach to treat PD patients.

### 3.2 Recommendation

Existing treatments that are available for PD tend to reduce or limit symptoms rather than cure the disease itself. With scientific development, new information about PD is emerging in the scientific world every day. In the light of increasing knowledge on PD, more promising treatment modalities will be of great help and may provide long‐lasting improvement in patients with PD.

Early diagnosis of PD and early detection are pivotal in the management of PD and may reduce its progression, therefore, enhancing the diseased person's life expectancy. This can be achieved by studying genomic makeup and the related epigenetic biomarkers and combining them with the clinical methods that are presently used for PD diagnosis; this can result in early detection and more accurate identification along with the disease protective therapies (Rathore et al., 2021; Uwishema et al., 2022). Therefore, targeting epigenetic mechanisms by drug therapies will provide a great therapeutic approach to PD treatment due to its specificity (Rathore et al., 2021; Uwishema et al., 2022). Moreover, it is considered that oxidative stress is a potential driver of the progression of PD at the cellular and physiological levels. Recent clinical trials have shown that administration of N‐acetylcysteine intravenously increases the rations of glutathione in blood and may consequently affect the dopaminergic system positively in PD patients. Also, N‐acetylcysteine is a promising multitarget prodrug due to its recently observed protective effects on astrocytes and oligodendrocytes (Silva et al., 2022). Furthermore, Leucine‐rich repeat kinase 2 (LRRK2) inhibition by different modes of inhibition such as kinase inhibitors is one of the most common therapeutic strategies. Targeting LRRK2 clinically is a straightforward option because the LRRK2 signaling induces kinase function as an outcome, which is also upregulated in all causing mutants PD. Inhibiting the LRRK2 kinase domain prevents endolysosomal deficits and also has neuroprotective effects which make it a promising treatment strategy (Wojewska & Kortholt, 2021). It is now known that future new therapies are working on replacing lost neurons in PD. The clinical trials with intrastriatal transplantation of human fetal mesencephalic tissue, rich in dopaminergic neurons, in PD patients revealed that cell replacement could work and, in some cases, induce major, long‐lasting improvement (Lindvall, 2015). In addition, α‐synuclein is a neuronal protein that regulates synaptic vesicle trafficking and neurotransmitter release. A clinical trial showed that treatments that use small molecule α‐synuclein aggregation therapy, monoclonal antibody, or gene therapy could be better than other clinical trials/therapies for the treatment in the future (Prasad & Hung, 2021). Moreover, a real‐life study revealed a continued reduction in motor fluctuations in patients who received 2 years of continuous treatment with apomorphine as an under‐the‐skin infusion (Meira et al., 2021).

## 4 CONCLUSION

As one of the neurodegenerative diseases, Parkinson's disease is a disease that affects people regardless of age worldwide. The clinical symptoms could be both motor and nonmotor, thus it is necessary to pay attention to symptoms even at an early age. The causes of the disease are various in terms of existing and new factors such as environmental and genetic factors, which is an issue demanding a lot of work to be done against the changes in both factors to overcome this disease. There is no absolute cure for the disease currently. While the existing options aim to reduce or limit the disease, there are future treatments scientists are working on, such as replacing lost neurons, α‐synuclein, and apomorphine. More clinical trials should be done to measure the efficacy of the other treatment modalities such as epigenetic modulation, LRRK2 inhibition, and intravenous administration of N‐Acetylcysteine as promising therapeutic strategies.

## FUNDING INFORMATION

None.

## CONFLICT OF INTEREST

The authors declare no conflicts of interest.

## AUTHOR CONTRIBUTION

**Olivier Uwishema**: Conceptualization, project administration, writing‐review and designing.

**Helen Onyeaka** and **Rawa Badri**: Reviewed and edited the first draft.

### PEER REVIEW

The peer review history for this article is available at https://publons.com/publon/10.1002/brb3.2577

## Data Availability

Not applicable.
